# Tuberculosis in Liberia: high multidrug-resistance burden, transmission and diversity modelled by multiple importation events

**DOI:** 10.1099/mgen.0.000325

**Published:** 2020-01-14

**Authors:** Mariana G. López, John B. Dogba, Manuela Torres-Puente, Galo A. Goig, Miguel Moreno-Molina, Luis M. Villamayor, Simeon Cadmus, Iñaki Comas

**Affiliations:** ^1^​ Tuberculosis Genomics Unit, Instituto de Biomedicina de Valencia (IBV-CSIC), Valencia, Spain; ^2^​ Tuberculosis and Brucellosis Laboratories, Department of Veterinary Public Health and Preventive Medicine, University of Ibadan, Ibadan, Nigeria; ^3^​ Center for Control and Prevention of Zoonoses, University of Ibadan, Ibadan, Nigeria; ^4^​ Tuberculosis Laboratory, National Public Health Reference Laboratory, National Public Health Institute of Liberia, Margibi, Liberia; ^5^​ Unidad Mixta “Infección y Salud Pública” (FISABIO-CSISP), Valencia, Spain; ^6^​ CIBER of Epidemiology and Public Health (CIBERESP), Madrid, Spain

**Keywords:** drug resistances, Liberia, TB transmission, tuberculosis, whole-genome sequencing, WGS

## Abstract

Tuberculosis (TB) surveillance is scarce in most African countries, even though it is the continent with the greatest disease incidence according to the World Health Organization. Liberia is within the 30 countries with the highest TB burden, probably as a consequence of the long civil war and the recent Ebola outbreak, both crippling the health system and depreciating the TB prevention and control programmes. Due to difficulties working in the country, there is a lack of resistance surveys and bacillus characterization. Here, we use genome sequencing of *Mycobacterium* *tuberculosis* clinical isolates to fill this gap. Our results highlight that the bacillus population structure is dominated by lineage 4 strains that harbour an outstanding genetic diversity, higher than in the rest of Africa as a whole. Coalescent analyses demonstrate that strains currently circulating in Liberia were introduced several times beginning in the early year 600 CE until very recently coinciding with migratory movements associated with the civil war and Ebola epidemics. A higher multidrug-resistant (MDR)-TB frequency (23.5 %) than current estimates was obtained together with non-catalogued drug-resistance mutations. Additionally, 39 % of strains were in genomic clusters revealing that ongoing transmission is a major contribution to the TB burden in the country. Our report emphasizes the importance of TB surveillance and control in African countries where bacillus diversity, MDR-TB prevalence and transmission are coalescing to jeopardize TB control programmes.

## Data Summary

1. Raw *Mycobacterium tuberculosis* read files have been deposited in the European Nucleotide Archive under the study accession number PRJEB32589, sample details are summarized in Table S1 (available with the online version of this article).

2. The reference *M. tuberculosis* complex (MTBC) common ancestor genome is available from https://doi.org/10.5281/zenodo.3497110.

3. The reference annotation genome of *M. tuberculosis* (strain H37Rv) is available from GenBank, accession number AL123456.2.

**Impact Statement**

Tuberculosis (TB) is still one of the top ten causes of death worldwide; despite the fact that Africa is the continent with the highest TB burden rate (308 cases per 100 000 population), scarce TB surveillance is conducted in most of its countries. Liberia is within the 30 countries with the highest TB burden and only 43 % of new cases are tested for drug resistance. The burden of infectious diseases in this West African country has increased alarmingly in the past 20 years as a consequence of the civil war and the Ebola outbreak, both crippling the health-care system and deteriorating TB programmes. Here, we describe the *Mycobacterium tuberculosis* population structure in Liberia, the genotypic drug-resistance (DR) frequency and the transmission rate. We reveal a worrying multidrug-resistant-TB burden together with new candidate resistance mutations not reported in available catalogues. We further demonstrate that strains currently circulating in Liberia were introduced to the country multiple times and from worldwide locations, probably promoting the outstanding diversity observed. Our results highlight the urgency for TB surveillance and control in African countries, where the sum of living situation, health-system condition and the biological particularities of the tubercle bacilli are likely favouring DR emergence and dispersion.

## Introduction

Liberia, on the West African coast, was founded at the beginning of the 19th century (around 1821) as a homeland for freed American slaves most of African ancestry. It is one of the few sub-Saharan countries devoid of colonial rule and considered the oldest African republic. Its population is composed of 95 % indigenous ethnic groups and a sizeable population of Lebanese, Indians and West African nationals. The country experienced 14 years of civil war that caused many deaths, family displacement and serious devastation of health facilities [1]. Consequently, poverty and the burden of infectious diseases, including tuberculosis (TB), malaria and human immunodeficiency virus/AIDS, dramatically increased. The Ebola outbreak in 2014 further crippled the health-care system, deteriorating the fragile TB prevention and control programmes [2, 3]. The entire scenario presumably exacerbated disease spread and emergence of multidrug-resistant (MDR)-TB strains.

Liberia is one of the three countries that have never conducted a drug-resistance (DR) survey; thus, MDR/extensively drug-resistant (XDR)-TB incidence is estimated based on data from neighbouring countries. The World Health Organization (WHO) also reports that fewer than 50 % of TB-positive cases have been tested, including for rifampicin resistance [4]. Consequently, the real burden of DR is largely unknown. Recently, it has been proved that DR prevalence at the country level can be accurately estimated by genomic surveillance [5]. Additionally, our increased knowledge of causative genetic variants demonstrates that first-line-drug susceptibility profiles can be precisely predicted and used at the patient level [6]. However, the sensitivity of WGS-DST (whole-genome sequencing – drug susceptibility testing) largely depends on the available catalogues of high-confidence mutations that have been mostly trained with samples from low-burden countries. In addition, DR variants may vary by geographical region and by the bacilli genetic diversity [7], hampering genome-based DST surveillance. Thus, it is unclear whether the sensitivity of WGS-DST will be compromised in Liberia and whether it can help to estimate the real burden of resistance.

We have carried out what is believed to be the first WGS study across two national TB referral hospitals in Liberia. To understand the origin of genetic diversity in the country, we compared the genomic data from 51 Liberian isolates with more than 9000 strains from all over the world and specifically from Africa. In addition, we identified MDR-TB strains based on published DR catalogues, as well as novel DR determinants. Overall, our results show a high epidemiological clustering rate, as well as a higher than expected MDR-TB percentage. This likely reflects the jeopardized public-health system after the devastating civil war and Ebola crisis.

## Methods

### Isolate collection and DST

The study was conducted during 2013–2016 at two referral hospitals, TB Annex Hospital (TBAH, Montserrado County, Liberia), the Ganta TB and Leprosy Rehabilitation Hospital (GTBLRH, Nimba County, Liberia), and other direct observation therapy(DOT) centres [1]. Following national guidelines, two sputum samples per patient were collected for smear microscopy and culture (with Lowënstein–Jensen medium). DST was done only for the two samples with new uncommon mutations in *katG* (LR60, LR69; see Results), using the proportion method [8] with modifications from the National Leprosy/TB Control Program, Ministry of Health and Social Welfare, Liberia, considering the following drug concentration breakpoints: 0.2 µg isoniazid (INH) ml^−1^, 1.0 µg INH ml^−1^, 40 µg rifampicin ml^−1^, 5 µg streptomycin ml^−1^, 2 µg ethambutol ml^−1^.

### WGS from heat-killed cultures

Culture isolates were inactivated by heating (100 °C, 30 min) at the National TB Reference Laboratory (Liberia) and shipped to a ﻿Biological Safety Level 3 (BSL3) laboratory in Valencia, Spain. After a second inactivation step (90 °C, 15 min), samples were used to prepare WGS libraries. Sequencing librarieswere constructed with a Nextera XT DNA library preparation kit (Illumina), following the manufacturer's instructions. Sequencing was performed on an Illumina MiSeq instrument.

### Bioinformatics analysis pipeline and identification of high-confidence DR mutations

Sequencing reads (300 bp paired-end) were trimmed with fastp [9]. kraken software [10] was used to remove non-MTBC (non-* Mycobacterium tuberculosis* complex) reads. Mapping and SNP calling were performed as described elsewhere [11, 12]. Briefly, MTBC reads were mapped to an inferred MTBC common ancestor genome (https://doi.org/10.5281/zenodo.3497110) using bwa [13]. SNPs were called with SAMtools [14] and VarScan2 [15], GATK HaplotypeCaller [16] was used for InDels calling. SNPs with a minimum of 10 reads (10×) in both strands and quality 20 were selected and classified based on their frequency in the sample as fixed (>90 %) or low frequency (10–89 %). InDels with less than 10× were discarded. SnpEff was used for SNP annotation using the H37Rv annotation reference (AL123456.2). Finally, SNPs falling in genes annotated as PE/PPE/PGRS, ‘maturase’, ‘phage’, ‘13E12 repeat family protein’; those located in insertion sequences; those within InDels or in higher density regions (>3 SNPs in 10 bp) were removed due to the uncertainty of mappings. Next, variants were compared with recently published catalogues with validated association between mutations and phenotypic resistance [17] in order to predict high-confidence resistance profiles to first- and second-line drugs. Lineages were determined comparing called SNPs with specific positions established [12, 18]. An in-house R script was used to detect mixed infections based on the frequency of lineage- and sublineage-specific positions. Briefly, specific lineage and sublineage SNP positions [18] were queried. If the lineage or sublineage variant frequency was below 90 %, then the sample was labelled as likely mixed infection (as described by Sobkowiak *et al*. [19]) (Supplementary File 1).

Multialignment files including the strains in the dataset were constructed including all the positions different from the MTBC ancestor, discarding invariant and annotated resistance positions. Pairwise distance was calculated with the R *ape* package and nucleotide diversity (π) with *pegas*. Only countries with more than 10 samples were considered when analysing diversity for each country, but all (465) samples were included in the continent (Africa) comparison. All graphics and statistical analysis were performed with R.

### Identification of novel candidate DR mutations

All mutations in DR-related genes and their regulatory regions but absent in common catalogues were inspected. Homoplasy, that is the occurrence of the same mutation somewhere else in the phylogeny, is a strong indicator of candidate DR mutations in MTBC [20]. In this regard, for each variant, homoplasy events were checked against a large reference dataset of 6146 genomes [21]. All variants, non-synonymous or InDels, present in at least two different lineages were selected for manual inspection. A bibliographical search was conducted in order to score mutations previously reported in the literature. We then assessed all the selected candidate mutations in the context of the susceptibility status for the rest of the drugs from the sample, since resistances are usually acquired sequentially [22].

### Phylogenetics, dating and geographical analysis

Phylogenies were reconstructed from fasta files with RAxML v8.2.11 [23] using the GTRCATI model and fast bootstrap. Tree visualization and editing were conducted in itol (https://itol.embl.de/). Dating analyses were performed by lineage including a representative dataset from available worldwide strains, i.e. 1184 L1 isolates, 2835 L2 and 5320 L4 [5, 11, 12, 18, 24–28]. Firstly, a neighbour-joining phylogeny was reconstructed with Mega7 [29], then data size was reduced with Treemmer [30] and finally maximum-likelihood (ML) phylogenies were reconstructed and used to estimate the time and geographical origin of each Liberian clade. Dating was performed with beast2 v2.5.1 [31], in all cases we used a log-normal distribution of the clock, coalescent constant population and a published substitution rate of 4.6×10^−8^ (0.20 SNPs per genome per year) as priors [32]. Markov Chain Monte-Carlo (MCMC) runs of 10M (L2) and 25M (L1 and L4) were replicated three times. Ancestral geographical origin was estimated using rasp [33] with the parameters previously proposed [26].

## Results

### Liberian TB burden is mainly caused by recent transmission

**Fig. 1.**
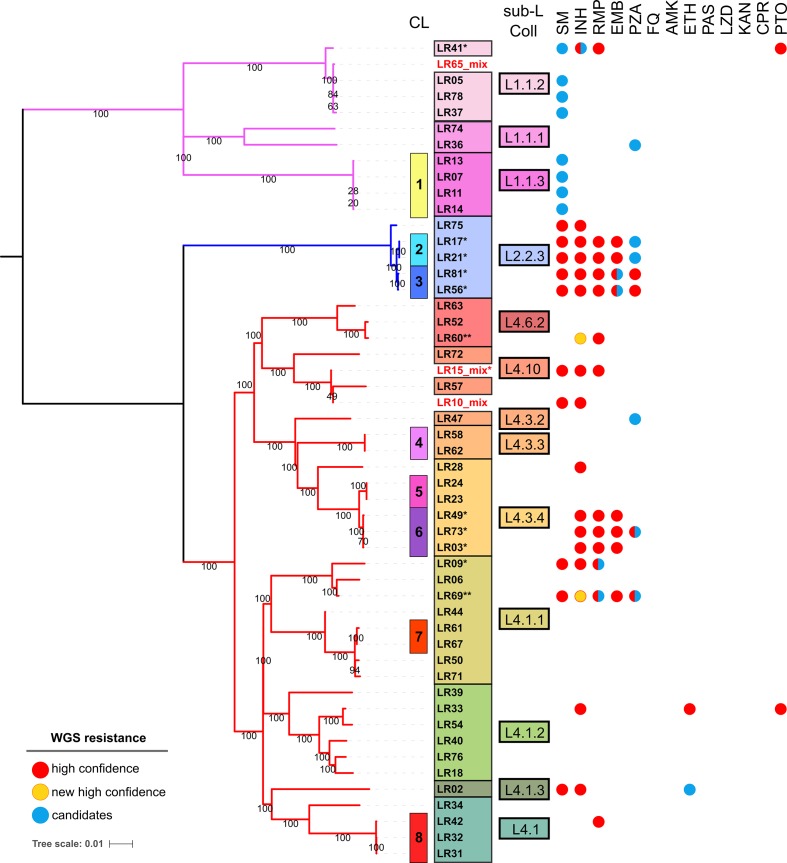
ML phylogeny displaying the diversity of Liberian strains, transmission clusters (CL) are numbered and indicated as coloured strips. Labels in red designate mixed infection isolates. Sublineages (sub-L) according to Coll *et al*. [18] are labelled and colour designated. High confidence (red circles) indicates resistance predicted with our in-house script based on curated catalogues; new high confidence (yellow circles) designates new mutations identified by manual inspection of variants and confirmed with phenotypic DST; candidates (blue circles) are new variants that are observed in resistance-associated genes, highly homoplastic or in the *pncA* gene, but not included in catalogues nor confirmed by phenotypic DST; half blue and half red circles indicate resistance and candidate mutations identified in the same strain. *, MDR strains; **, multidrug resistance identified with the new high-confidence mutations. AMK, Amikacin; CPR, capreomycin; EMB, ethambutol; ETH, ethionamide; FQ, fluoroquinolones; INH, isoniazid; KAN, kanamycin; LZD, linezolid; PAS, para-aminosalicylic acid; PTO, prothionamide; PZA, pyrazinamide; RMP, rifampicin; SM, streptomycin. Scale bar indicates mean number of nucleotide substitutions per site

A ML phylogeny was reconstructed including 51 samples (6994 concatenated SNPs, Fig. 1). L4 was the most frequent lineage, followed by L1 and L2. All L1 strains belonged to L1.1 and Beijing L2 were classified as L2.2.3 or Asia Ancestral L3 group [34, 35]. Four L4 sublineages were identified, being the most common 4.1.1 (lineage X) and 4.3.4 (Latin American-Mediterranean -LAM- with RD174, Fig. 1). Additionally, three samples were determined as mixed infection, all of them involving one *M. tuberculosis* strain and one *Mycobacterium africanum* lineage (L5 or L6).

TB transmission was evaluated by performing clustering analysis from pairwise distance and also considering cluster monophyly. Three different thresholds of median distance were considered in order to establish the timeframe of the transmission [36]: 15, 12 and 5 SNPs (see Methods for details). The clustering rates were 39 % for the two higher cut-offs, including 20 strains grouped in 8 clusters; a value of 35 % was obtained for the 5 SNPs level for which CL_7 was excluded with a distance of 6 SNPs. Interestingly, cluster size didn’t change for the different cut-offs evaluated (Fig. 1, Table S2), indicating transmission occurred at a similar time span for all members within the cluster. Thus, our data show that most transmission events were within a timeframe of 10 years [36], suggesting that ongoing transmission, rather than reactivation from older infection, is disproportionately contributing to the Liberia TB burden.

### Resistance profile predictions from WGS suggest a high burden of MDR-TB

Inspecting available catalogues of high-confidence resistance mutations, we predicted 33 fully susceptible strains, 5 poly-resistant, 2 rifampicin monoresistant, 1 INH monoresistant and 10 MDR cases (Fig. 1, Table S1). We also identified 15 novel mutations not described before, but falling in well-documented DR genes. We consider those mutations as candidate resistance mutations. As expected for DR mutations in *M. tuberculosis *, many of them are homoplastic [20]. Novel mutations in *pncA* are now considered by the WHO as very likely pyrazinamide-resistance variants. Thus, the data suggest that the novel candidate mutations are indeed DR-associated variants, although no DST was available to confirm our prediction.

Among the candidate variants, most confer resistance not only to pyrazinamide and streptomycin, but also to ethambutol or ethionamide (Table 1); notably, they occurred in the three different lineages retrieved in Liberia. In the case of rifampicin, both resistance mutations in *rpoB* (S441Q, R827C) were identified in isolates with an additional high-confidence *rpoB* mutation. For INH, we identified two non-synonymous variants and a 1 bp deletion in *katG*. Mutation P232S co-occurred with another high-confidence mutation in *katG*. On the contrary, the D142A variant [37] and the 1 bp deletion were of special interest as the carrier strains (LR60, LR69) also harbour high-confidence rifampicin-resistance mutations. As those mutations are not included in available catalogues, a phenotypic DST analysis was performed in both isolates confirming resistance to INH. Including these new high-confidence variants, our prediction of MDR cases increased to 23.5 % (12 isolates, Fig. 1).

**Table 1.** List of newly identified mutations

Gene	Genomic position	Nucleotide change	Mutation	Isolate name	Occurrence*	Homoplasy†	Variant frequency (%)	Drug
*embB* Rv3795	4 249 583	G/A	D1024N	LR56	17	3+	100	EMB
				LR81			97.44	
*ethA* Rv3854c	4 327 378	G/T	Y32STOP	LR02	1	2	100	ETH
*gid* Rv3919c	4 407 816	GC/G	1 bp del	LR07	10	3+	100	SM
				LR11			100	
				LR13			100	
				LR14			100	
	4 407 982	A/G	L74S	LR41	4	3+	100	
	4 408 148	C/G	A19P	LR05	1	2	99.04	
				LR37			100	
				LR78			100	
*katG* Rv1908c	2 155 418	G/A	P232S	LR41	3	3	29.31	INH
	2 154 624	GC/G	1 bp del‡	LR69	–	–	100	
	2 155 687	T/G	D142A‡	LR60	–	–	100	
*pncA* Rv2043c	2 288 782	T/C	R154G	LR73	2	2	38.52	PZA
	2 289 115	G/A	H43Y	LR47	–	–	100	
	2 289 199	C/A	E15STOP	LR17	–	–	100	
				LR21	–	–	100	
	2 289 202	A/C	C14G	LR69	3	2+	95.24	
	2 289 210	T/C	N11S	LR36	–	–	100	
*rpoB* Rv0667	761 127	T/C	S441Q	LR69	1	2	100	RMP
	761 128	C/A		LR69			100	
	762 285	C/T	R827C	LR09	1	2	8.75	

*Occurrence indicates the number of isolates from the reference dataset where the mutation appeared.

†Homoplasy indicates the number of lineages where the mutation is observed and + means that it also appeared in different sublineages.

‡High-confidence mutations confirmed by DST phenotypic analysis.

EMB, Ethambutol; ETH, ethionamide; INH, isoniazid; PZA, pyrazinamide; RMP, rifampicin; SM, streptomycin.

Finally, we explored compensatory mutations and found *rpoC* V483A [20] compensating for *rpoB* S450L in two strains belonging to the same transmission cluster (LR17, LR21). Likewise, we found two independent mutations at the *oxyR–ahpC* intergenic region (positions −39 and −9) associated with the new *katG* deletion (LR60) and D142A (LR69), respectively. There is strong evidence that the *ahpC* mutations area associated with rare *katG* mutations as is the case for the two novel *katG* mutations found in the Liberian dataset [38, 39].

### Dating and geographical analysis suggest common links of L1 and L2 strains to South-East Asia

**Fig. 2.**
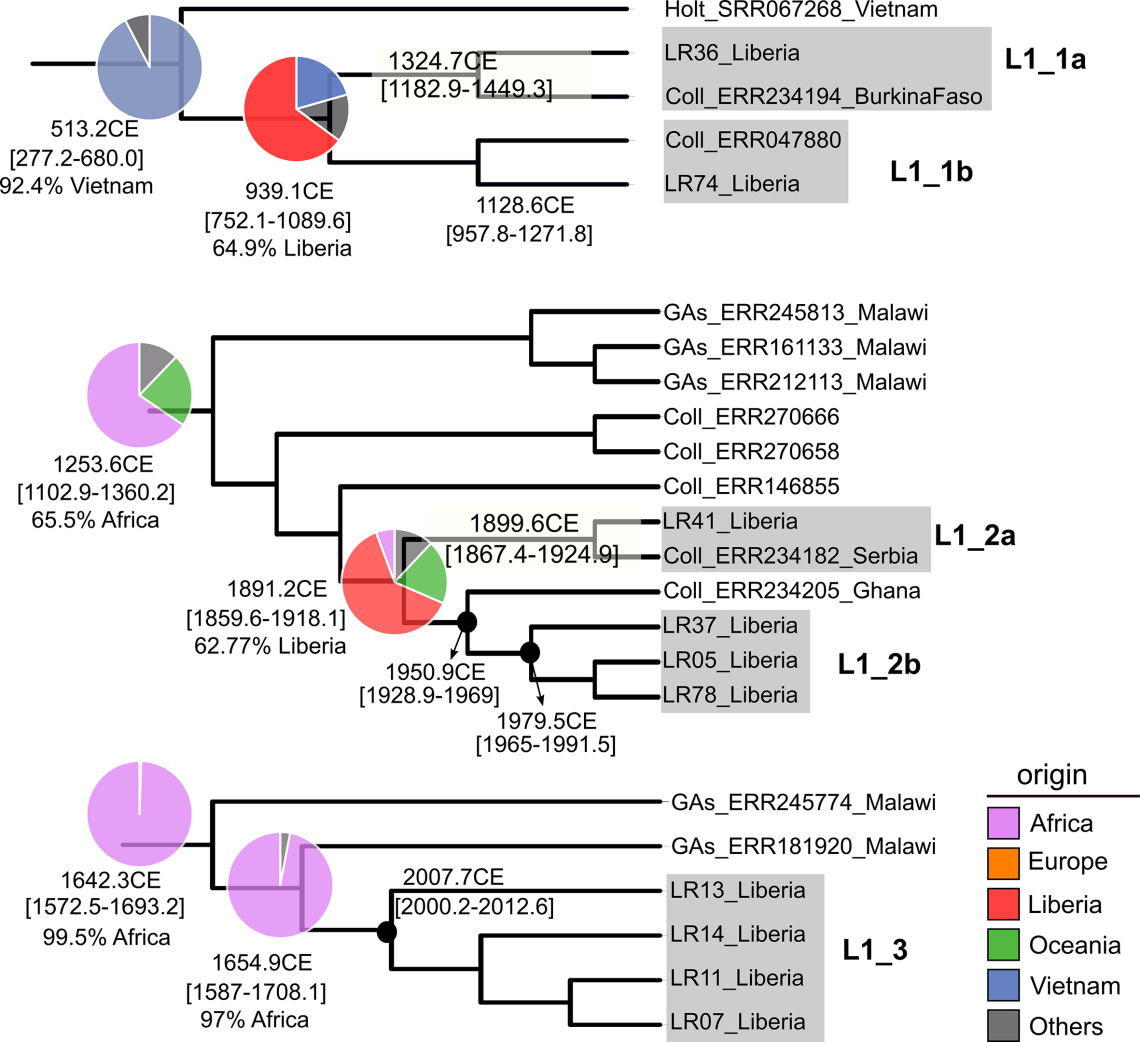
Liberian clades from global lineage 1 phylogeny (see Fig. S1) including Beast dating results. tMRCA median values and 95 % HPD (highest posterior density) intervals are indicated in years CE. Pie charts represent rasp results indicating the geographical origin of ancestors as shown in the key. Isolate names include run accession number, first author name from the reference publication and patient country of origin. GAs, Guerra-Asunção.

Origins and dating of Liberian L1 and L2 strains were estimated using a global phylogeny representing lineage diversity (Fig. S1) and recognizing the limited sampling in our dataset. According to beast dating analyses, we identified three clades that were either older (before the 19th century), contemporaneous (around the 19th century) or much more recent than Liberia foundation (1821). The oldest L1 clade was L1_1 (subclades L1_1a and L1_1b) with an estimated age around 1100 CE (Fig. 2). Surprisingly, the most probable origin was placed in Vietnam (92.4 %). L1_2, also with two subclades, displayed a most recent common ancestor (MRCA) dating back to the late 19th century, compatible with its introduction during foundation. The youngest L1_3 clade has a MRCA in the 21st century, consistent with these strains belonging to the same transmission cluster. According to the phylogeographical analysis, L1_3 is included in a larger African cluster, but diverged from the closest reference strains around 1655 CE (Fig. 2).

**Fig. 3.**
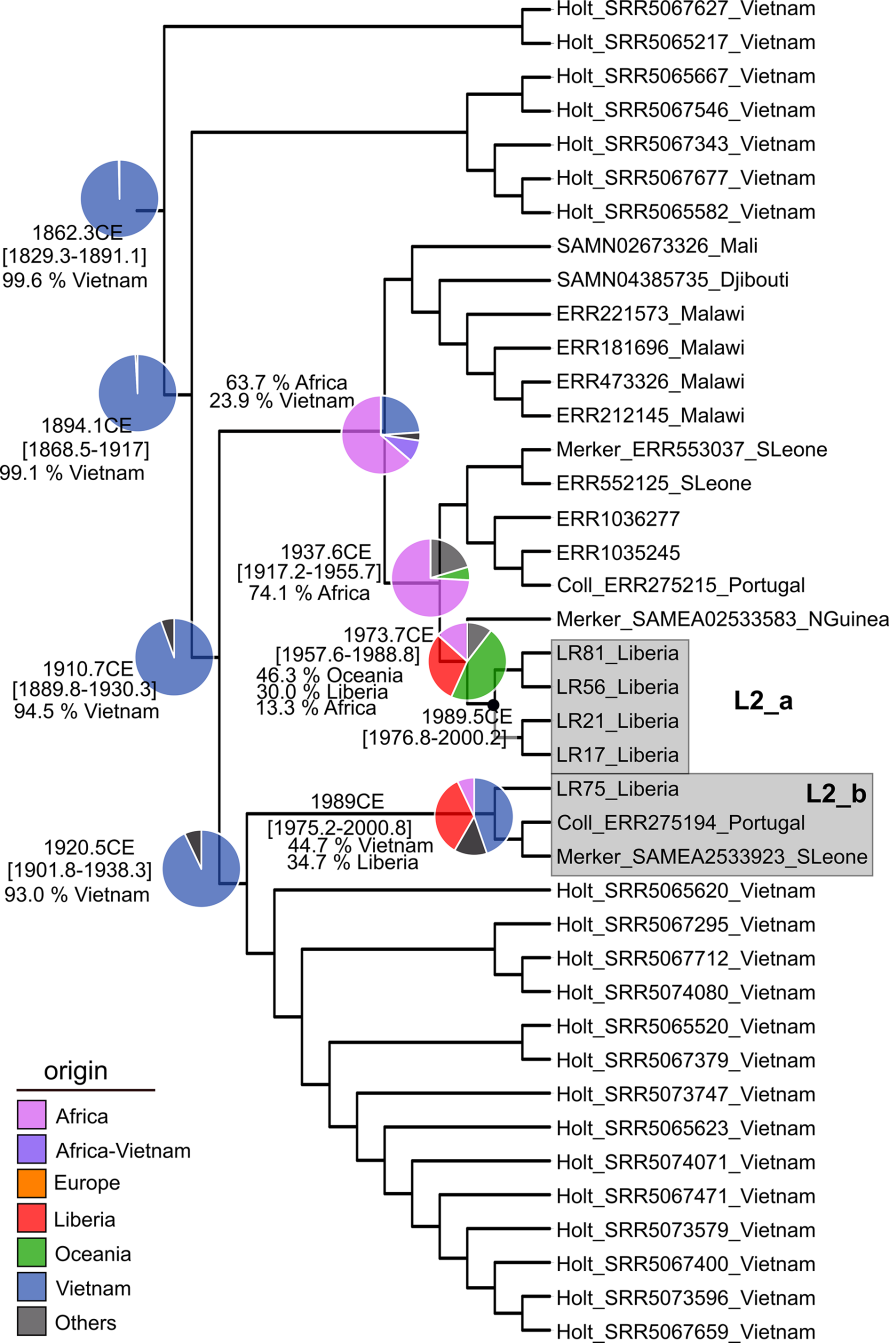
Liberian clade from global lineage 2 phylogeny (see Fig. S2) including beast dating results. tMRCA median values and 95 % HPD intervals are indicated in years CE. Pie charts represent rasp results indicating the geographical origin of ancestors as shown in the key. Isolate names include run accession number, first author name from the reference publication and patient country of origin

Contrary to L1, L2 revealed more recent origins. A global phylogeny showed that Liberian strains were grouped in two different subclades (L2_a, L2_b) with a time to the most recent common ancestor (tMRCA) in the middle-late 20th century. Phylogeographical analysis placed the MRCA of both subclades in Africa but an older Vietnamese origin was evidenced, yet posterior to Liberia foundation, (Figs 3 and S2).

### Liberia shows the highest diversity indexes in Africa for L4

**Fig. 4.**
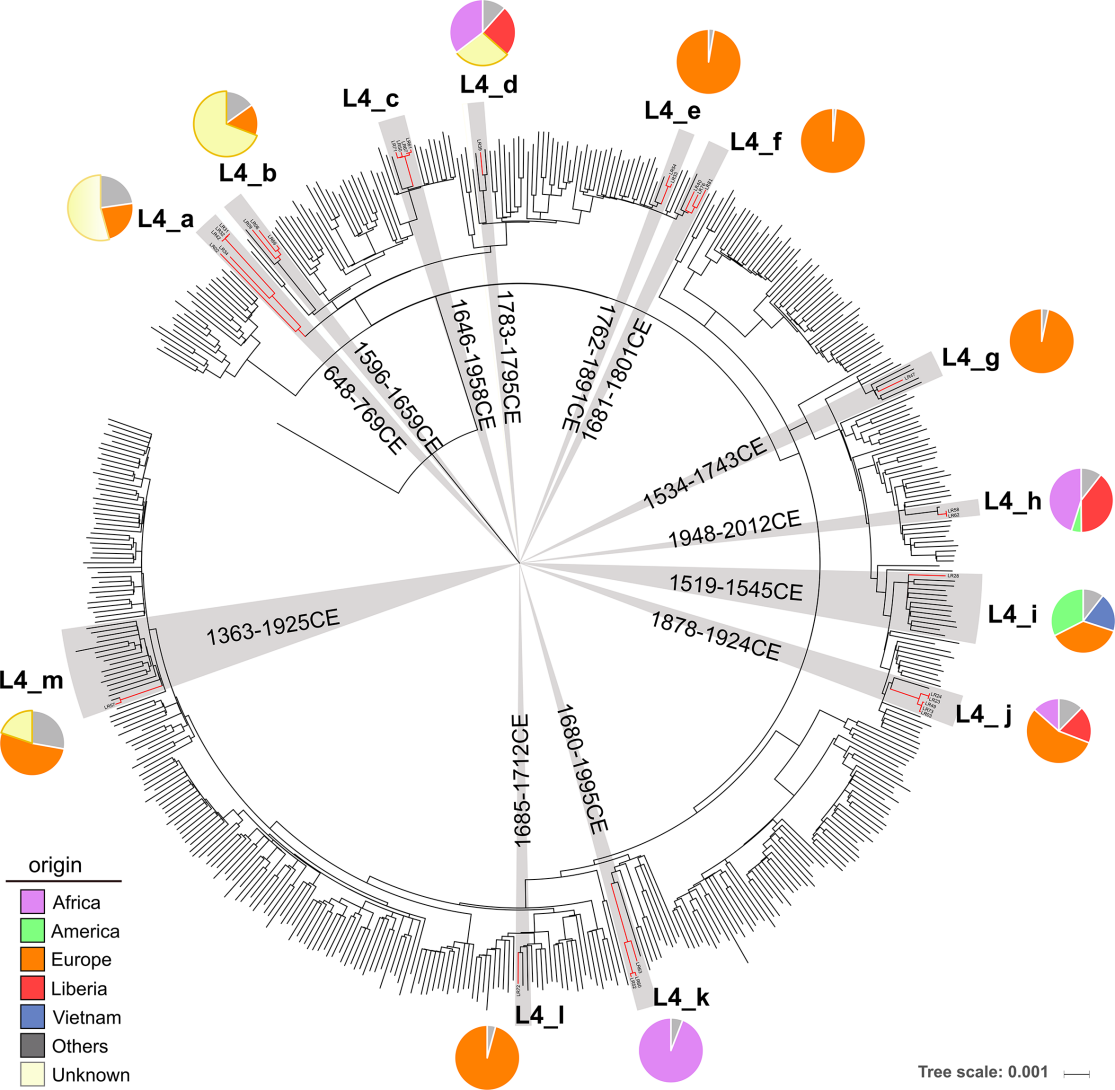
Global lineage 4 phylogeny including a total of 525 worldwide strains representing all sublineages (4.1 to 4.6 and 4.10). Multiple sequence alignment with 66 630 SNPs was used to reconstruct the ML phylogeny. Liberian clades are highlighted. beast dating results: tMRCA median values intervals are indicated in years CE. Pie charts represent rasp results indicating the geographical origin of ancestors as shown in the key. Grey shading designates nodes considered for dating, red lines denote Liberian samples. The mean substitution rate recovered from beast analysis was 0.21 SNPs per genome per year (0.17–0.27, 95 % HPD). Fig. S3 contains detailed rasp and beast results. Scale bar indicates mean number of nucleotide substitutions per site.

Liberian strains clustered together with other worldwide samples except for L4_k, which formed a distinct clade (Fig. 4). Dating performed on L4 revealed three general situations resembling L1, but on a larger scale. Most of the Liberian clades were older or close to the time of country foundation (clades a, b, d, f, g, i and l, (Figs 4 and S3). The tMRCA of the oldest clade L4_a was estimated between 648 and 769 years CE. The estimated tMRCA range of all the other clades can be grouped between 1500 and 1800 years CE approximately. All ancient clades had a most probable origin in Europe or Europe/America as described for the lineage. The exception is L4_d in which Africa appeared as the most plausible geographical origin, suggesting that the ancestor of that clade was circulating in Africa before the foundation of Liberia.

By contrast, L4_h and L4_j appeared as recent Liberian clades. The tMRCA was estimated between years 1948 and 2012 CE for L4_h; with the most probable geographical origin placed in Africa. The ancestor for L4_j was dated between 1879 and 1924 CE with the origin located in Europe/Africa. The remaining four clades (L4_c, L4_e, L4_k, L4_m) displayed a wide dating range and, thus, were difficult to assign to a specific period. For these clades, Europe is also the most probable place of origin except for L4_k, with African origin, corresponding to the endemic sublineage Cameroon L4.6.2 [12].

**Fig. 5.**
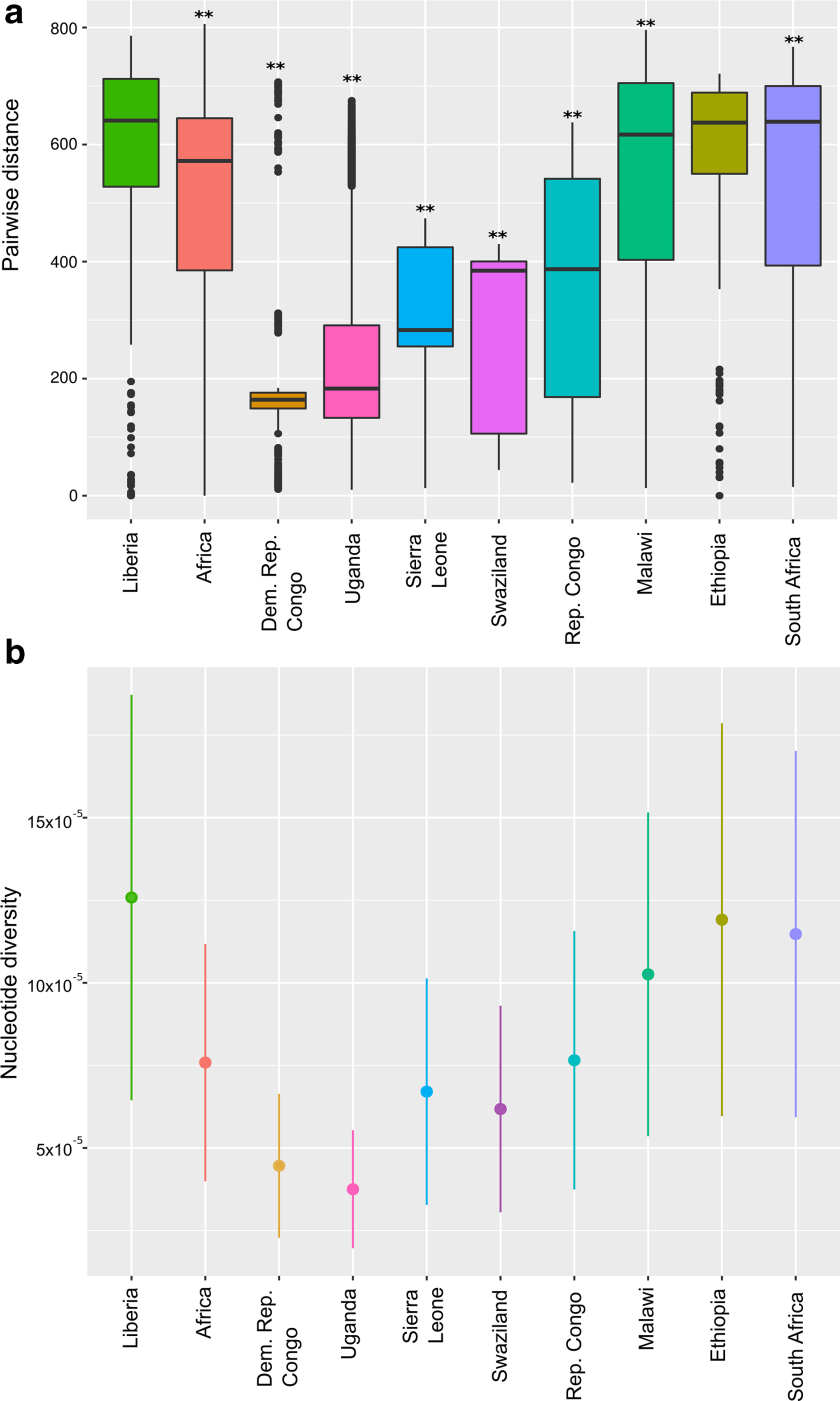
Lineage 4 genetic diversity for African countries and the whole continent. (a) Box plot of pairwise distances, asterisks indicate significant differences from Liberia (Wilcoxon rank-sum test; Table 2). (b) Nucleotide diversity per site (π), error bars indicate sd.

The distribution of the strains in so many clades and various dating results, suggests that Liberia has an unusual diversity for L4. We then compared pairwise genetic distance and nucleotide diversity among Liberian samples to L4 diversity observed in other African countries (Table S3) and in the continent as a whole. Liberia displayed the highest diversity estimated for both parameters (Fig. 5). In this regard, differences were significant when compared both to the pool of all African countries and also to the individual level (Table 2) except Ethiopia, for which great genetic L4-MTBC diversity has already been reported [40]. Since very few samples from other lineages were obtained in Liberia, we only evaluated L4 diversity but L1 also appeared dispersed in different clades and mixed with non-Liberian strains, suggesting that its diversity could also be elevated.

**Table 2.** Summary of genetic diversity measurements for different regions

Region	No. of samples	Nucleotide diversity (π)	π sd	Mean pairwise distance	*P* value*
Liberia	51	12.58×10^−5^	6.13×10^−5^	584.12	
Africa	465	7.58×10^−5^	3.59×10^−5^	509.55	*P* <0.0001
Democratic Republic of the Congo	33	4.46×10^−5^	2.18×10^−5^	209.06	*P* <0.0001
Ethiopia	19	11.9×10^−5^	6.00×10^−5^	563.01	*P*=0.0596
Malawi	84	10.26×10^−5^	4.90×10^−5^	545.19	*P* <0.0001
Republic of the Congo	12	7.66×10^−5^	3.91×10^−5^	355.62	*P* <0.0001
Sierra Leone	14	6.71×10^−5^	3.43×10^−5^	308.48	*P* <0.0001
South Africa	45	11.48×10^−5^	5.54×10^−5^	536.58	*P* <0.0001
Swaziland	16	6.18×10^−5^	3.12×10^−5^	286.01	*P* <0.0001
Uganda	172	3.75×10^−5^	1.79×10^−5^	246.96	*P* <0.0001

*P value corresponds to Wilcoxon rank-sum test for comparisons with Liberia.

## Discussion

Liberia is within the 30 countries with the highest TB burden, with a rate of 308 cases per 100 000 population [4] and a devastated health system. Despite this, no surveillance on DR is available for this country and estimates are based on extrapolations from neighbouring countries. Additionally, the diversity of the TB bacillus is unknown and transmission assessment is scarce. We have conducted what is believed to be the first report using WGS with the aim of providing valuable insights on these three fronts even with a limited sampling.

Lineage assignment from WGS revealed a prevalence of L4, as was reported for the whole of Africa [41]. Surprisingly, all L2 strains belong to the L2.2.3 sublineage mentioned as generally uncommon in Africa [42] but frequently observed in a recent study [34]. Unexpectedly, *M. africanum* was only found in mixed infections, despite its high frequency in neighbouring countries [43]. This result suggests that these lineages are underdetected in culture media, as has been reported elsewhere [44].

Because of the wide dating range and different origins estimated, Liberia strains were introduced not only with the slaves who founded the country, but also from different waves of migrants after and before the country's foundation, and this trend still continues. For each lineage, different situations were observed. Results suggest that L1.1.2 and L1.1.3 appeared in Liberia after the arrival of the freed slaves (Fig. 2, L1_2, L1_3). In agreement, rasp results suggest that the ancestors of both Liberian sublineages were circulating in Africa before the country’s foundation, reinforcing the scenario of a recent introduction from the vicinity. In contrast, L1.1.1 (Fig. 2, L1_1) strains were older than the others and without evidence of African predecessors. L1.1.1 strains have a strong link with Vietnam, remarkably related to a distinct L1 clade endemic to this country [25]. Surprisingly, L2 strains also show a recent ancestry link with Vietnam. Results are in agreement with previous reports [34], which proposed that one of the latest Beijing introductions to Africa occurred in the 20th century, coming from East Asia. Although, a potential link between Asia and South Africa was suggested [45], this is unlikely the origin of Liberian strains as they have no clear link with the southern country; thus, our results point to additional introductions from Asia. In this regard, the presence of Chinese and probably Taiwanese people associated with mineral resource exploitation in Liberia is well known. Also, a high proportion of recent Indian and Lebanese immigrants to Liberia have been documented.

In the case of L4, it is noticeable that with only one African exception (L4_d), all older clades are deeply linked to Europe and to a lesser degree to America. This is in agreement with reports of the origin of the lineage [11, 18, 46]. By contrast, younger Liberian L4 clades were closely related to Africa, suggesting that introduction to Liberia was directly from the continent and posterior to the foundation of the country. It is remarkable that L4 pairwise distances among Liberian strains were statistically higher than for the whole continent, even when Africa was postulated as the MTBC place of origin and, thus, concentrating the highest genetic diversity [11, 32, 47]. We concluded that the enlarged variability estimated in Liberia results from the combination of the ancient African diversity [40, 41, 48] and the multiple recent importations, most of them within a timeframe of 20 years.

According to the WHO, only 43 % of new TB cases in Liberia are tested for rifampicin resistance. Thus, estimates of the MDR-TB burden in Liberia are based on extrapolations from neighbouring countries with similar epidemiological profiles. Our prediction based on WGS-DST projected a worrying high percentage of MDR (23.5 %, 12/51 cases) in the modest dataset analysed. Additionally, our comprehensive sequence inquiry allowed us to identify two additional variants in *katG* not included in widely used WGS resistance catalogues. Both mutations conferred INH resistance confirmed by phenotypic DST, reclassifying carrier strains as MDR. We also found a large diversity of new candidate DR mutations in agreement with the enlarged diversity observed. Cumulatively, the current DR scenario most likely has been precipitated by the fractured public-health system in Liberia after years of civil war. A situation that is further exacerbated by the Ebola crisis, which undermined previous attempts at improving patient care in the country [2]. Thus, many patients were left untreated or were not able to adhere to treatment; therefore, likely generating a highly diverse DR mutational spectrum. Altogether, these findings suggest that in high burden countries, with underperforming TB control programmes, unknown DR mutations could be more common and hampering WGS-based diagnostics [7].

Transmission was also high and associated with DR. We observed a 39 % clustering rate in a small dataset of 51 samples, with three out of eight clusters involving MDR strains. Cluster analysis revealed recent transmission events including all identified lineages. Phenomena extrinsic to the pathogen, like host malnutrition and deficiency in the health system, can be powerful drivers of TB transmission [49], supporting the virulence and capacity to persist of the pathogen [50]. In the specific case of Liberia, the civil war and the Ebola outbreak generated a scenario of large population movements (within and outside the country) and devastated the public-health environment [1, 2].

In conclusion, our results highlight a great MDR-TB burden, a substantial ongoing transmission and an outstanding genetic diversity circulating in Liberia. Most of these phenomena probably have been fuelled by the dwindling public-health infrastructure during the Ebola crisis and the associated people movement to/from neighbouring countries. Phylogenetic dating also suggests a continuous influx of new genetic diversity in the region before and after the foundation of the country. However, we don’t have available detailed epidemiological information about the patients and there is only a limited number of genomes from neighbouring countries. We believe that if these data were available, they would have helped to a great extent to provide further insights into the roles of recent conflict and the Ebola crisis. Finally, this report emphasizes the urgency for TB surveillance and control in African countries, where the interaction among human living conditions, health systems and the biological particularities of the tubercle bacilli are likely favouring DR emergence, involving novel mutations, and dispersion.

## Data bibliography

1. DNA sequences generated as part of this study have been deposited at the European Nucleotide Archive under study accession number PRJEB32589 (Table S1) (2019)

2. Camus J.C., Pryor M.J., Medigue C, Cole S.T. The reference genome of Mycobacterium tuberculosis (strain H37Rv) was obtained from GenBank, accession number AL123456.2 (https://www.ncbi.nlm.nih.gov/nuccore/AL123456.2/) (2002)

## Supplementary Data

Supplementary material 1

Supplementary material 2
