# Supplementary material for: Tuberculosis in Liberia: high multidrug-resistance burden, transmission and diversity modelled by multiple importation events
Source: Microb Genom. 2020 Jan 14;6(1):e000325. doi: 10.1099/mgen.0.000325 (PMC7067037; doi:10.1099/mgen.0.000325)
Supplement: Supplementary material 1 [file mgen-6-325-s001.pdf]

Figure S1. ML phylogeny of 270 global strains of Lineage 1. It was constructed from a multi sequence alignment of 48,691 concatenated SNPs. Liberian clades detailed in Figure 2 are highlighted. Beast dating analysis resulted in a mean substitution rate of 0.25 SNPs per genome per year (0.22-0.27, 95% HPD).

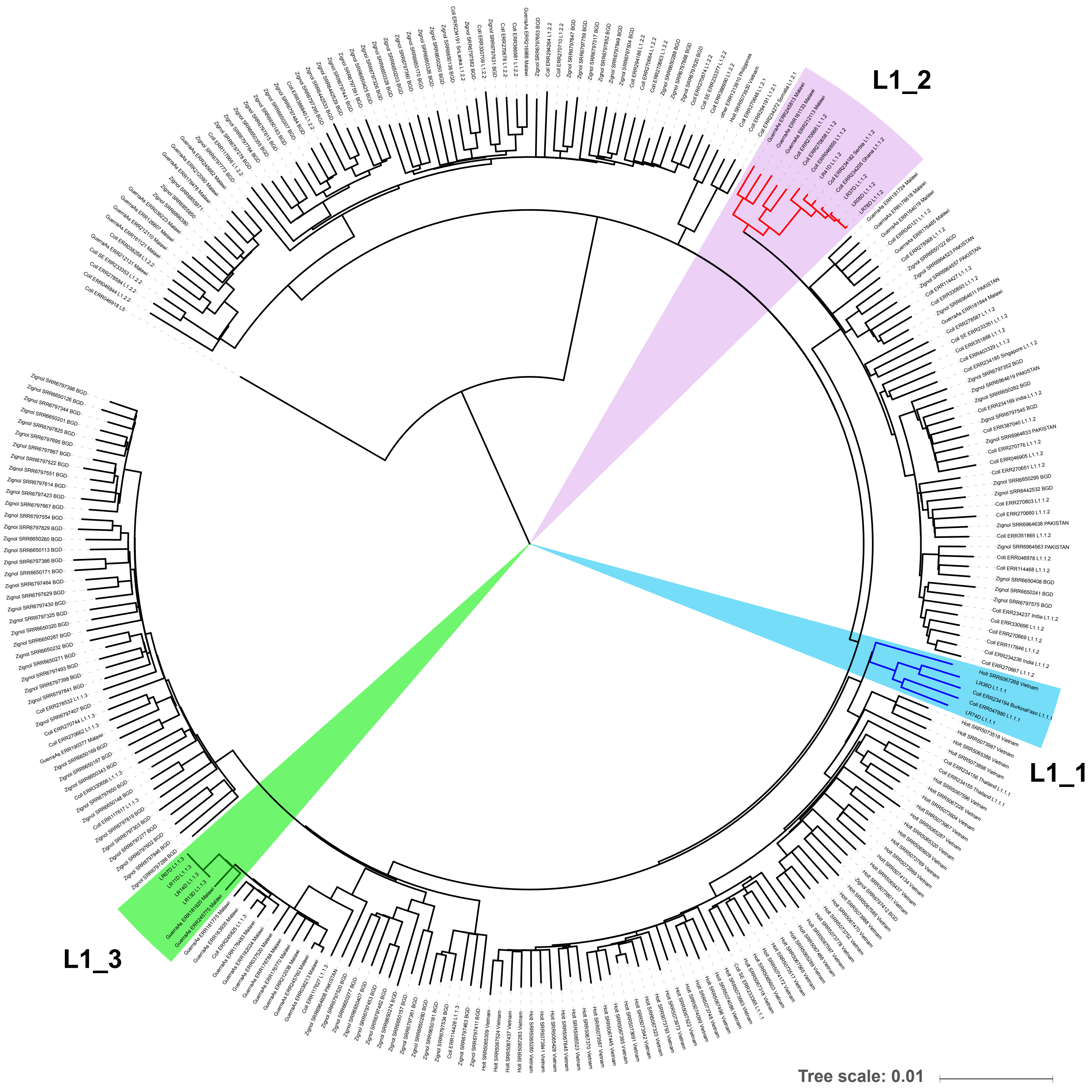

Figure S2. ML phylogeny of 303 global strains of Lineage 2, it was constructed from a multi sequence alignment of 24,740 concatenated SNPs. Liberian clade detailed in Figure 3 is highlighted. Beast analysis revealed a mean mutation rate of 0.23 SNPs per genome per year (0.20-0.26, 95 % HPD)

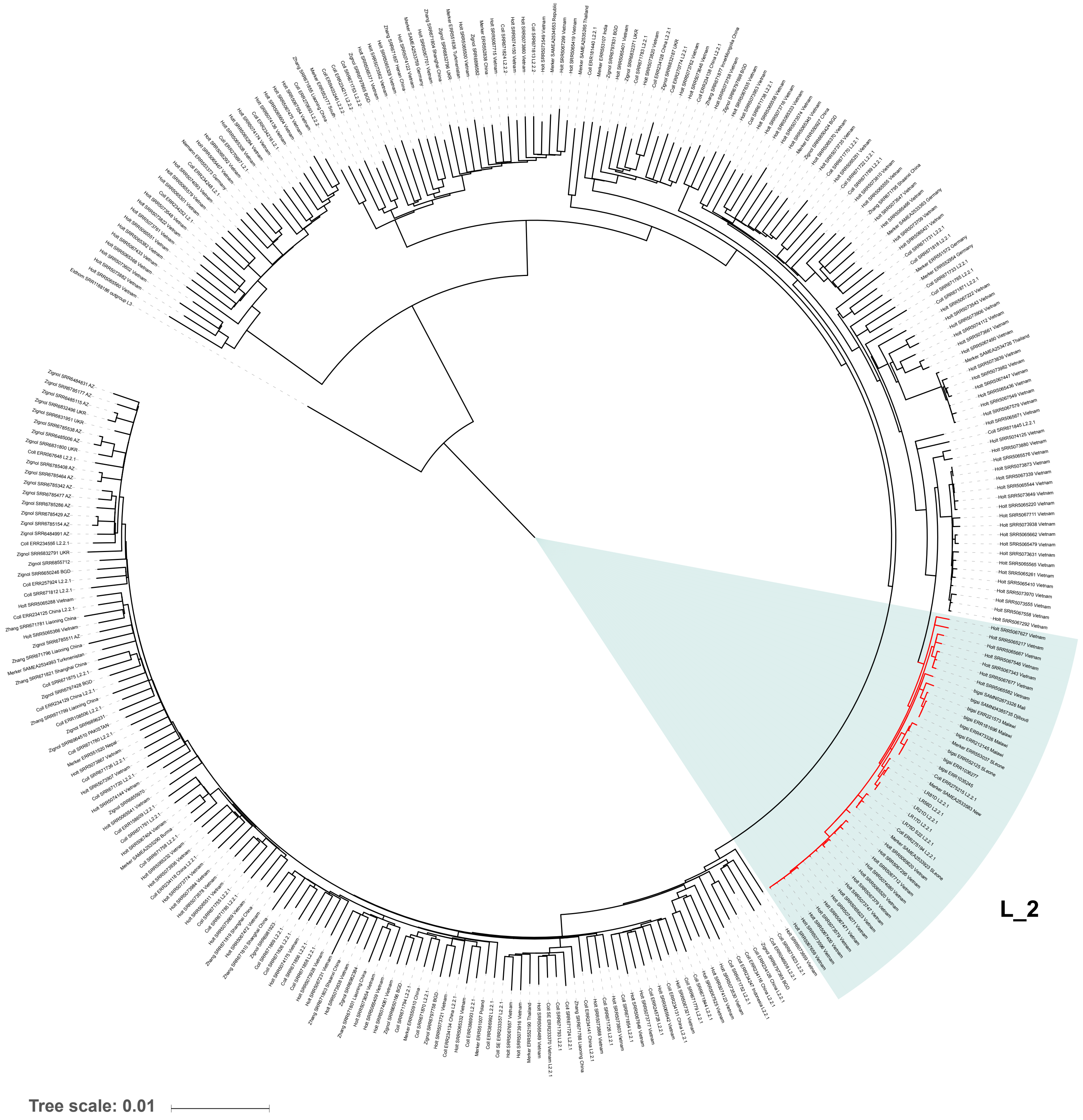

Figure S3. Dating and phylogeographical analysis of Liberian clades indicated in Figure 4. Beast dating results, tMRCA median values and 95 % HPD interval are indicated in years CE. Pie charts represents RASP results indicating geographical origin of ancestors as referenced in legend

	range	95% HPD	origin
**L4_a**	647.6 - 769.4CE	[271.7-957.4] [415-1050.2]	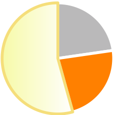 23% Europa
**L4_b**	1595.6 - 1658.9CE	[1464.4-1710.6] [1541.1-1754]	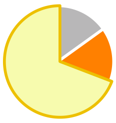 15.7% Europa
**L4_c**	1646.5- 1958.2CE	[1495.1-1662] [1935.1-1976.4]	unknown
**L4_d**	1782.9- 1795.4CE	[1743.1-1813.9] [1756.1-1827.9]	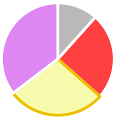 35.4% Africa 25% Liberia
**L4_e**	1762.4- 1891.5CE	[1692.2-1815.6] [1844.1-1931.5]	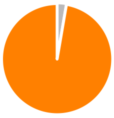 97.3% Europa
**L4_f**	1681.1- 1800.9CE	[1692.2-1815.6] [1844.1-1931.5]	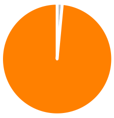 98.5% Europa
**L4_g**	1534.1- 1743.2CE	[1404.1-1645.4] [1656.7-1816.2]	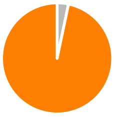 96.7% Europa
**L4_h**	1948.1- 2011.8CE	[1917.8-1971.9] [2004.5-2014]	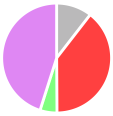 45.1% Africa 39.4% Liberia
**L4_i**	1519.5- 1545.3CE	[1403.8-1611.4] [1436.2-1638.2]	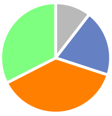 37.5% Europa 32.4% America
**L4_ j**	1878.5- 1923.7CE	[1841.4-1908.5] [1890.6-1951.8]	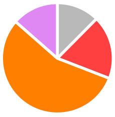 55.6% Europa 18.5% Liberia 13.5% Africa
**L4_k**	1679.6- 1995.2CE	[1581.4-1754.8] [1982.4-2005.3]	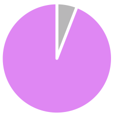 94.1% Africa
**L4_l**	1685.2- 1711.7CE	[1603.5-1752.9] [1635.3-1774.9]	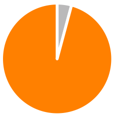 95.8% Europa
**L4_m**	1362.9- 1924.8CE	[1212.1-1489.3] [1887.4-1956.6]	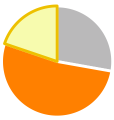 52.4% Europa

origin
- Africa
- America
- Europa
- Liberia
- Vietnam
- Others
- Unknown

File S1. Script to search for linage and mixed infections (Rscript)

```
#A list with the lineages and sublineages specific SNPs is requires named
snp_phylo.csv [argument 1] with lineage name and genomic position of variant,
also .snp files separated by columns including the genomic position of the snps
"Position", reference and alternative nucleotides and frequency of the variant
"VarFreq"
#to run the script: Rscript mixed_infections.R snp_phylo.csv path_to_snp_files
#output is phylogeny_result

library(IRanges)

args=commandArgs(trailingOnly = TRUE)
phylo_pannel <- read.delim(args[1], header = TRUE, stringsAsFactors=FALSE)
#defining the specific snps file
snp_file = list.files(args[2],pattern="\\.snp$") #defining the files for
evaluate mixed infections

#output creation
phylogeny_result=data.frame('Sample'=character(),'Phylogeny'=character(),
'mixed_infection'=character(),check.names = FALSE, stringsAsFactors = FALSE)


#Lineage identification
  snp_file$VarFreq=sub("%","",as.character(snp_file$VarFreq)) #eliminate the %
symbol if it is present in the .snp files
  snp_filo=which(is.element(phylo_pannel$Position,snp_file$Position)) #searching
lineage and sublineages specific snps in .snp files

for(i in 1:length(archivos)) #para todos los snp.final
{
  if (length(snp_filo)==0) {phylogeny_result$Phylogeny[i] = "Unknown" #if none
specifis snp is idenfied the lineage is assigned as unknown
  } else
{phylogeny_result$Phylogeny[i]=phylo_pannel[snp_filo[length(snp_filo)],'lineage'
]} #the script select the deepest sublinaege i.e. L4.3.2.1 instead of L4

#If more than one specific snp is found with frequency lower than 90% the sample
is defined as mixed_infection

phylo_coinfec_snps=snp_file[which(is.element(snp_file$Position,phylo_pannel$Posi
tion) & snp_file$VarFreq<90),] #searching for snps with frequencies lower than
90%
  if (dim(phylo_coinfec_snps)[1]==0)
{phylogeny_result[i,'mixed_infection?']="No"
  } else {
    phylogeny_result$`mixed_infection?`[i]=""
    for(j in 1:dim(phylo_coinfec_snps)[1])
    {phylogeny_result$`mixed_infection?
`[i]=paste(phylogeny_result$`mixed_infection?
`[i],paste(phylo_coinfec_snps$Position[j],paste(phylo_coinfec_snps$VarFreq[j],"%
",sep=""),phylo_pannel$lineage[which(phylo_pannel$Position==phylo_coinfec_snps$P
osition[j])],sep = ","),sep = " | ")}
    snp_filo_main <-
phylo_pannel[which(is.element(phylo_pannel$Position,snp_file$Position[which(snp_
file$VarFreq>90)])),]

    phylogeny_result[i,'mixed_infection?']<-
tryCatch({phylogeny_result[i,'mixed_infection?']=paste(paste(snp_filo_main$Posit
ion[dim(snp_filo_main)
[1]],paste(snp_file$VarFreq[which(snp_file$Position==snp_filo_main$Position[dim(
snp_filo_main)[1]])],"%",sep=""),snp_filo_main$lineage[dim(snp_filo_main)
[1]],sep = ","),phylogeny_result$`mixed_infection?`[i],sep = " | ")}, warning=
```

```
function(w){return("Error!")}, error= function(e){return("Error!")}) #If more
than two snps appeared in the same position it gives error
    phylogeny_result[i,'Phylogeny']="mixed_infection"
  }
}

write.table(result, file="phylogeny_result",row.names = FALSE, quote=FALSE,
sep='\t')
```
